# Validation of the Persian version of the inflammatory bowel disease questionnaire (IBDQ) in ulcerative colitis patients

**Published:** 2015

**Authors:** Iradj Maleki, Tarang Taghvaei, Maryam Barzin, Kamyar Amin, Alireza Khalilian

**Affiliations:** 1 Head, Inflammatory Diseases of Upper GI Tract Research Center,; 2 Mazandaran University of Medical Sciences, Sari, Iran.; 3 Department of Radiology, Mazandaran University of Medical Sciences, Sari, Iran.; 4 Department of Social Medicine, Mazandaran University of Medical Sciences, Sari, Iran.

**Keywords:** Inflammatory Bowel Disease, Quality of life, Ulcerative Colitis, Persian, Linguistic validation

## Abstract

**Background:**

Inflammatory bowel diseases (IBD) are a group of inflammatory conditions of the colon and small intestine that may have critical consequences on patient’s quality of life (QOL). Many disease-specific QOL tools have been developed recently. The McMaster Inflammatory Bowel Disease Questionnaire (IBDQ) is one of them. The aim of this study was to translate the IBDQ from English to Persian and evaluate the validity and reliability of this version of the McMaster IBDQ.

**Methods:**

68 subjects with ulcerative colitis were recruited in this study. The original IBDQ was translated into Persian using back- translation method. The reliability of the subscales and the summary score of the Persian IBDQ was demonstrated by intraclass correlation coefficients, their validity was evaluated by their correlations with SF-36, visual analogue scale and colitis activity index.

**Results:**

All dimensions of IBDQ met the standards of construct validity and were correlated well with SF-36, visual analog scale and colitis activity index. IBDQ was able to discriminate the different groups of patients. The intraclass correlation coefficient was very high and its value was close to one (P<0.05). All dimensional scores differed significantly between the baseline and the follow-up measurement.

**Conclusion:**

The findings of this study conclude that the Persian translation of IBDQ confers satisfactory psychometric and cultural properties when applied to a sample of Iranian population with inflammatory bowel disease. This questionnaire is recommended for use in clinical trials and in the assessment of efficacy of interventions and therapy.

Inflammatory bowel disease (IBD) is a group of inflammatory conditions of the colon and small intestine. The major types of IBD are ulcerative colitis and Crohn's disease , characterized by an alternating course with unpredictable recurrences despite lifelong treatment (1, 2). Disease course is associated with many complications including surgical interventions, drug side effects and many psychological factors (3), that may have critical consequences on the patient’s quality of life (1, 3). Quality of Life (QOL) has been defined as the subjective evaluation of life as a whole or the patient's appraisal and satisfaction with their current level of functioning compared with what they perceive to be possible or ideal (4).

Many general measurement tools exploring health status and disease activity in IBD had been developed in the past decades (2, 5), but none of these measures were able to assess the effects of IBD on the patients QOL (2). Recently, there has been a clear trend towards more frequent use of disease-specific instruments (1). Many disease-specific QOL tools have been developed in North America, including the McMaster Inflammatory Bowel Disease Questionnaire (IBDQ), which has been the most widely used in the world (1, 5). The inflammatory bowel disease questionnaire (IBDQ) is the standard instrument for the assessment of QOL that was developed by Guyatt et al. that is appropriate for use both in interviews and as a self-administered questionnaire (6, 7). It is a valid and reliable questionnaire in a clinical setting and sensitive to change during a period of time (5). There is a little information about the quality of life of IBD patients in Iran. Also, the IBDQ has not been translated into Persian. The aim of this study was to evaluate the various aspect of validity and reliability of the Persian translation of the IBDQ.

## Methods

**Patients: **In this study, 68 patients with ulcerative colitis (UC) were recruited from Imam Khomeini Hospital, Sari, Iran. The diagnosis of UC was made according to the Lennard-Jones criteria (8). The exclusion criteria were presence of a psychological disorder, malignancy, chronic diseases affecting the quality of life such as diabetes mellitus, heart and respiratory disease, cirrhosis, physical disability renal failure and ileostomy. 

The participants were asked to complete the IBD questionnaire twice, at an interval of 6-8 weeks. Before the beginning of study, the interviewer explained the purpose of survey to all eligible individuals and requested their participation. The individuals were informed that participating in the study was not compulsory. Informed consent was obtained, and the patient data were kept fully anonymous.

**Questionnaire: **The original IBDQ is an interviewer administered, disease-specific questionnaire for evaluating QOL (7). It consists of 32 items, which are divided into 4 domains: bowel related symptoms, systematic symptoms, emotional function, social function (2, 3, 7, 9-11). Responses to each item are scored in a 7-point scale in which 1 indicates worst function and 7 the best (2, 5, 12). The total IBDQ points range from 32 to 224, with a higher score reflecting better quality of life (3, 13).

The IBDQ was translated into Persian using back- translation method. First, the questionnaire was translated by one gastroenterologist. Second, an official bilingual translator did a back translation into English. Then, the back-translation was compared with original questionnaire with a reasonable match. Finally, the questionnaire was pretested in a small group of patients with IBD to determine whether it was understandable and easy to complete (4).

The following questionnaires were used for validation:

**Visual analogue scale** **(VAS): **The VAS was used as a measure to evaluate the degree of bowel function and the state of general well-being experienced by the patients themselves. It is a horizontal Likert scale that measures from 0 to 10. Zero indicates the least favorable health state and 10, the most favorable health state, with a crossline representing the middle point (3, 7, 9).

**Short Form Health Survey** **(SF-36): **SF-36 is a non- disease specific instrument to assess all the domains of health status by means of 36 items. This questionnaire was categorized into 8 domains: physical function, role limitations due to physical health problems, bodily pain, general health perception, energy and vitality, social function, role limitations due to emotional problems, and mental health. A higher score indicates a better health status. This questionnaire has been validated and used in many countries and various medical conditions. The Persian version of SF-36 has been recently validated (14) and used in the study. 

**Colitis Activity Index** **(CAI): **Disease activity was assessed by CAI. The CAI was scored according to the parameters of frequency of diarrhea, nocturnal diarrhea, visible blood in stool, fecal incontinence, abdominal pain, general well-being, abdominal pain and the need for anti-diarrheal drugs. The total score ranged from 0 to 21, with remission defined as a score of below 10 (15).

**Assessing Validity (Construct validity and discriminant ability): **Construct validity indicating the correlation between the equivalent domain scores of the Persian IBDQ, VAS and SF-36 and was analyzed using the Spearman correlation coefficient. Discriminant validity examines the capability of IBDQ to differentiate between groups of interest. In this study, the patients’ perception of well-being according to VAS was categorized into two groups. VAS score ≤ 4 was considered as unwell and ≥ 4 was considered as well. Discriminant validity was calculated using the Mann-Whitney U test.

**Reliability: **The reliability of questionnaire refers to the consistency of domain scores when carried out repeated measures (3, 9). In this study, the patients with no significant changes in symptoms were considered as patients with stable disease and completed the Persian IBDQ within 2 months of the initial questionnaire. The reliability of IBDQ was assessed using the intraclass correlation coefficient (ICC). An ICC of 0.5 indicates adequate reliability and an ICC of 0.8 indicates excellent reliability (5). Sensitivity to changes indicates the ability of the questionnaire to distinguish any clinically important changes over time, if it seems small (9). Sensitivity to change was evaluated by comparing the mean of IBDQ score and score change within the patients who reported a change in symptoms between the 2 completions and over time using a paired t-test.

All tests were two-sided, and were considered to be statistically significant at p<0.05. Statistical analysis was performed with SPSS software (Version 13.0).

## Results

A total of 68 UC patients were recruited in the study of which 25(36.7%) and 43 (63.3%) cases were males and females, respectively. The mean age of patients was 40.4±14.28 years. 

**Construct Validity:** The Spearman’s correlation coefficients were calculated to assess the association between the 4 IBDQ domain scores and the VAS for general well-being, the total SF-36 and the CAI for disease activity. The positive and significant correlation was seen between the domains of IBDQ and VAS total score. The range of coefficients varied between 0.69-0.84. Most correlations between IBDQ domains and SF-36 total score were strong, except bowel symptoms which showed a much lower correlation than other domains. The observed associations between IBDQ domains and disease activity index were moderately high and negative and varied from -.0376 to -0.701.

**Discriminant Validity: **The subjects who participated in the study were categorized according to VAS into 2 groups: well (VAS≥4) and unwell (VAS<4). The results of comparisons between 2 groups are shown in table 2. There was a significant difference for all four dimensions of IBDQ between both groups.

**Table 1:** Construct Validity of Persian IBDQ

**IBDQ dimension**	**SF-36** **(Total score)**	**VAS**	**CAI**
Bowel symptoms	0.610	0.724	-0.701
Systemic symptoms	0.750	0.769	-0.519
Emotional Function	0.863	0.697	-0.376
Social Function	0.775	0.840	-0.630

All correlations were significant and p-value was less than 0.05.

**Table 2:** Discriminant validity of Persian IBDQ

**IBDQ dimension**	**Unwell**	**Well**	**Pvalue**
Bowel symptoms	30 (4.58)	53 (9.16)	<0.001
Systemic symptoms	15 (3)	25.17 (5.02)	<0.001
Emotional Function	39.3 (6.11)	56 (12.65)	<0.001
Social Function	13.3 (6.11)	27.17 (5.79)	<0.001

**Reliability: **A subgroup of 20 patients was asked to complete IBDQ and CAI after 6-8 weeks. 12 patients reported stable status during the course of the study. For assessing the reliability of Persian IBDQ, these patients completed the questionnaire again. Reliability outcomes are shown in table 3. Intraclass correlation coefficients in four domains were very high, close to one and there was no statistical difference between two measurements.

**Sensitivity to Change: **Sensitivity to change was analyzed in patients who showed a change in disease activity between two assessments. The results of paired t-test are shown in table 4. Four IBDQ domains had highly significant differences between the baseline and the follow-up scores.

**Table 3:** Reliability of Persian IBDQ

	**Bowel ** **symptoms**	**Systemic symptoms**	**Emotional Function**	**Social Function**
Test	51.66	23.91	53.33	26
Retest	52.16	23.91	53.33	25.58
Mean Difference	-0.5	0	0	0.416
Intraclass Correlation Coefficient	0.98	0.99	0.99	0.99
P-value	<0.0001	<0.0001	<0.0001	<0.0001

**Table 4:** Sensitivity to Change of the IBDQ

	**Bowel symptoms**	**Systemic symptoms**	**Emotional Function**	**Social Function**
Test	41.71	20.85	50.71	23.28
Retest	57.14	27.57	61.42	28.57
Mean Difference	15.42	6.71	10.71	5.28
Percent of changes	37	32	21	23
P-value	<0.05	<0.05	<0.05	<0.05

## Discussion

The findings of this study show that the Persian version of IBDQ is a valid and reliable tool for assessing quality of life in IBD patients in Iran, which indicate that it can be used in clinical research. There was some missing data in this study indicating that this questionnaire is acceptable and intelligible for patients. 

An interview-based method was chosen for this study. The main advantage of this method is the reduction of incomplete questions and missing data (5). Although some studies have used a self-administered methods (1, 3, 16), but the results provided by these two methods have no significant differences (5, 17). The validation procedure in this study consists of assessment of construct and discriminant validity, reliability and sensitivity to change. The high correlation between IBDQ and SF-36, VAS and CAI showed that all dimensions of IBDQ met the standards of construct validity. Similar findings were seen in some cross-cultural adaption studies (5, 11, 18). 

As shown in details in this study, IBDQ was able to discriminate between groups of patients with different perception of general well-being according to VAS. These results are comparable with other studies (3, 5, 7, 9). The reliability of the IBDQ was tested by test–retest with an interval of 6-8 weeks. The intraclass correlation coefficient was very high and close to one. Considering that intraclass correlation coefficient above 0.8 is generally accepted and satisfactory (12), these results indicate that Persian IBDQ has an excellent reliability. These findings are very close to those obtained in other validated translations (1-3, 11), except in the study conducted in Sweden (10) that reported poorer reliability. This could be due to a longer interval between the two tests in the study mentioned above. Because of uncontrollable factors such as disease activity of coexisting diseases or change in general well-being due to other psychosocial factors such as working conditions or family life in this interval may reduce the reliability of the study. Sensitivity to change was assessed by comparing the four IBDQ dimensional scores in patients who reported change between the first and second assessment. All dimensional scores differed significantly between the baseline and the follow-up measurement. In general, the results were very acceptable and comparable with other translations (3, 5, 7, 9). The limitation of this study was a lack of standardization of IBDQ in other subgroups such as patients with Crohn's disease and its complications. In order to overcome the limitation of the study, a multicentric study with a larger number of patients including both Crohn's disease and ulcerative colitis with a variety of their complications are recommended.

The findings of this study conclude that the Persian translation of IBDQ has satisfactory psychometric and cultural properties when applied to a sample of Iranian patients with inflammatory bowel disease. This questionnaire is recommended for use in clinical trials and in the assessment of efficacy of interventions and therapy.

## Notes

**Funding:** The authors would like to thank the Deputy for Research of Mazandaran University of Medical Sciences for the grant and the support for this research.
